# Vivisection

**Published:** 1880-02

**Authors:** 


### VIVISECTION.

At the last meeting of the Erie County Medical Society, resolutions were presented by Dr. James P. White, and unanimously adopted, opposing measures, which have been brought forward in the Legislature, tending to limit the privilege now enjoyed of employing living animals for experimental purposes.

The subject referred to in these resolutions is one of no small importance to the profession and to the public. We only regret that in this issue we can not review the many benefits arising from vivisection, and contrast them with the narrow-minded quixotic spirit which actuates the bill now pending in the Legislature.

The great advantage of experimental study are so apparent to all intelligent physicians, that for them the claims of vivisection need no advocate. But it behooves them, as it does the medical press of the State, to exert all the influence that can be brought to bear, to defeat the passage of a bill which would be so prejudicial to the advancement of medical science, and therefore so detrimental to the public.

